# Increased levels of a mycophenolic acid metabolite in patients with kidney failure negatively affect cardiomyocyte health

**DOI:** 10.3389/fcvm.2024.1346475

**Published:** 2024-03-06

**Authors:** Eva Harlacher, Corinna Schulte, Sonja Vondenhoff, Philippe Schmitt-Kopplin, Philippe Diederich, Christian Hemmers, Julia Moellmann, Julia Wollenhaupt, Rogier Veltrop, Erik Biessen, Michael Lehrke, Björn Peters, Georg Schlieper, Christoph Kuppe, Jürgen Floege, Vera Jankowski, Nikolaus Marx, Joachim Jankowski, Heidi Noels

**Affiliations:** ^1^ Institute for Molecular Cardiovascular Research (IMCAR), RWTH Aachen University, Aachen, Germany; ^2^ University Hospital RWTH Aachen, Aachen, Germany; ^3^ Research Unit Analytical BioGeoChemistry, Helmholtz Zentrum München, Neuherberg, Germany; ^4^ Analytical Food Chemistry, Technical University of Munich, Freising, Germany; ^5^ Department of Internal Medicine I, Cardiology, University Hospital RWTH Aachen, Aachen, Germany; ^6^ Department of Pathology, Cardiovascular Research Institute Maastricht (CARIM), Maastricht University, Maastricht, Netherlands; ^7^ Aachen-Maastricht Institute for Cardiorenal Disease (AMICARE), RWTH Aachen Campus, Aachen, Germany; ^8^ Department of Nephrology, Skaraborg Hospital, Skövde, Sweden; ^9^ Department of Molecular and Clinical Medicine, Institute of Medicine, Sahlgrenska Academy at the University of Gothenburg, Gothenburg, Sweden; ^10^ Division of Nephrology and Clinical Immunology, University Hospital RWTH Aachen, Aachen, Germany; ^11^ Department of Biochemistry, Cardiovascular Research Institute Maastricht (CARIM), Maastricht University, Maastricht, Netherlands

**Keywords:** chronic kidney disease, cardiovascular disease, uremic toxin, cardiomyocyte, drug metabolite, mycophenolate mofetil

## Abstract

Chronic kidney disease (CKD) significantly increases cardiovascular risk and mortality, and the accumulation of uremic toxins in the circulation upon kidney failure contributes to this increased risk. We thus performed a screening for potential novel mediators of reduced cardiovascular health starting from dialysate obtained after hemodialysis of patients with CKD. The dialysate was gradually fractionated to increased purity using orthogonal chromatography steps, with each fraction screened for a potential negative impact on the metabolic activity of cardiomyocytes using a high-throughput MTT-assay, until ultimately a highly purified fraction with strong effects on cardiomyocyte health was retained. Mass spectrometry and nuclear magnetic resonance identified the metabolite mycophenolic acid-β-glucuronide (MPA-G) as a responsible substance. MPA-G is the main metabolite from the immunosuppressive agent MPA that is supplied in the form of mycophenolate mofetil (MMF) to patients in preparation for and after transplantation or for treatment of autoimmune and non-transplant kidney diseases. The adverse effect of MPA-G on cardiomyocytes was confirmed *in vitro*, reducing the overall metabolic activity and cellular respiration while increasing mitochondrial reactive oxygen species production in cardiomyocytes at concentrations detected in MMF-treated patients with failing kidney function. This study draws attention to the potential adverse effects of long-term high MMF dosing, specifically in patients with severely reduced kidney function already displaying a highly increased cardiovascular risk.

## Introduction

Chronic kidney disease (CKD) with a prevalence of 13.4% worldwide and of 10.6% for advanced CKD stages 3–5 has developed to become a serious health problem and is one of the world's fastest growing causes of death (1, 2). As the damage of kidneys increases, reflected by the decreasing glomerular filtration rate (GFR), metabolic waste substances and uremic toxins gradually accumulate in the blood, negatively impacting cell physiology and thereby contributing to the diseased state of the patient (3). In patients reaching CKD stage 5, kidney replacement therapy becomes necessary in the form of dialysis or kidney transplantation.

Around 50% of patients with CKD stage 4–5 suffer additionally from cardiovascular disease (CVD) and both the risk of cardiovascular events as well as disease prognosis depend on the severity of kidney impairment (4, 5). Even independent from classical cardiovascular risk factors, the risk of CVD in CKD is high, making CKD an independent risk factor for CVD (6). Ultimately, CVD accounts for 40%–50% of all deaths in patients with CKD stage 4–5, compared with 26% in controls with healthy kidney function (7, 8). Several uremic toxins have been associated with increased morbidity and mortality in patients with CKD, and specifically also with increased cardiovascular risk and mortality (9–13). However, it is expected that additional circulating mediators may contribute to increased cardiovascular morbidity in patients with CKD, although currently they remain undiscovered.

We thus performed a screening for potential novel mediators of reduced cardiovascular health within the dialysate obtained after dialysis of patients with CKD. In this report, we describe the identification of a drug metabolite that exerts a negative impact on the health of cardiomyocytes at levels reported in patients with CKD treated with the drug.

## Methods

### Fractionation of dialysate using chromatography

As a pool of a broad spectrum of uremic toxins in blood and with potential negative impact on cardiomyocyte health, 500 L hemodialysate solution (“dialysate” for short) was collected as waste fluid after hemodialysis (Fresenius Genius90 System) of 10 patients with CKD stage 5, as approved by local authorities of the University RWTH Aachen (Germany) (EK/196/18). The uremic toxins present in the dialysate after hemodialysis were concentrated over reversed-phase (RP) chromatography and then, using a stepwise approach and different chromatographic methods, separated into different fractions for analyzing potential effects on cardiomyocytes.

#### Reversed-phase (RP) chromatography

Preparative reversed-phase chromatography (RPC) with a C18 column [LiChroprep®RP-18 (40–63 µm); Merck] and a stepwise gradient of ethanol in water (20%, 40%, 60%, and 100%) was used to concentrate and fractionate the dialysate into four initial fractions. The fractions were lyophilized, solved in 5 ml ddH_2_O, and used in cell culture experiments at a dilution of 1:1000. A uremic toxin pool consisting of all four fractions, and thereby representing the full spectrum of the toxins present in the dialysate, was used in a dilution of 1:100.

#### Size-exclusion chromatography

The “20% dialysate fraction” obtained after preparative reversed-phase chromatography was further fractionated using size-exclusion chromatography (SEC) (S100; Sephacryl S100 high Resolution; GE Healthcare). In total, 72 fractions of 0.9% NaCl (each 4 ml) were obtained, pooled in groups of two, tested for effects on cardiomyocytes in a 3-(4,5-dimethylthiazol-2-yl)-2,5-diphenyl-2H-tetrazolium bromide (MTT) assay at a dilution of 1:4 in comparison with vehicle control-treated cells.

#### Anion- and cation-exchange chromatography

Fractions 37–50 from the size-exclusion chromatography were pooled, desalted by analytical reversed-phase chromatography (Chromolith® Performance RP-18 endcapped 100–4.6; Merck), and fractionated in two steps based on their charge. First, samples were buffered with a 20-mM K_2_HPO_4_ solution (pH 8.0), loaded onto an anion-exchange chromatograph (Uno® Q12 Monolith Anion-Exchange Column; Bio-Rad), and stepwise eluted by increasing concentrations of NaCl in 20-mM K_2_HPO_4_ solution (pH 8.0). Next, after pH adjustment to pH 3.5, the flow-through of the anion-exchange chromatography was loaded on a cation-exchange chromatograph (Uno® S12 Monolith Cation Exchange Column; Bio-Rad) and stepwise eluted with increasing NaCl concentrations in 20-mM K_2_HPO_4_ solution (pH 3.5). The flow-through was designated as neutral substances. All fractions were desalted over the analytical reversed-phase chromatography, lyophilized, and dissolved in 50 µl ddH_2_O before testing for effects on cardiomyocytes in an MTT assay at a dilution of 1:20.

#### Reversed-phase chromatography with a continuous gradient

“Anionic fraction 1” and the “neutral fraction” were further fractionated using reversed-phase chromatography with a continuous gradient (0%–30% ethanol in water). After lyophilization, fractions were dissolved in 100 µl ddH_2_O, pooled in groups of two, and tested for effects on cardiomyocytes in an MTT assay at a dilution of 1:8.

### Fourier transform ion cyclotron mass spectrometry (FTICR-MS)

We previously described bioactivity-guided chromatography combined with ultrahigh-resolution mass spectrometry for the discovery of novel bioactive compounds (14, 15). Here, the dried bioactive and inactive chromatographic fractions were solubilized in minimal volume of water (200 µl) diluted in methanol 1:5 (v:v) for direct injection at 2 µl/min into electrospray ionization negative mode ultrahigh-resolution mass spectrometry. Briefly, we used a Bruker Solarix 12 Tesla Fourier transform ion cyclotron resonance mass spectrometer (FTICR-MS) located at the Helmholtz Zentrum, Munich, Germany. The resolution (>400,000 at m/z 400) and the mass error (<0.2 ppm) were sufficiently precise to compute the exact molecular formulae from the elements (C, H, O, N, S, X). Further, the mass 495.15174 was isolated and fragmented in collision-induced dissociation (CID) with 2, 5, and 7.5 eV, showing a neutral mass loss of a sugar unit (C_6_H_8_O_6_).

### Nuclear magnetic resonance (NMR) sample preparation and measurement

A total of 54 µl of the dried and reconstituted (in H_2_O) bioactive fraction was spiked with 6 µl D_2_O containing sodium 3-(trimethylsilyl)propionate-d4 (2 mM) as a chemical shift reference and di-sodium hydrogen phosphate (1.5 M, pH 7) to buffer the sample at pH 7. The sample was analyzed in a 1.7 mm nuclear magnetic resonance (NMR) tube. Experiments were carried out on an 800 MHz Bruker AVANCE lll spectrometer equipped with a 5 mm QCI-probehead at 300 K. One-dimensional (1D) 1H spectra were recorded using a 1D version of the nuclear overhauser effect (NOE) experiment (noesypr1d) with on-resonance presaturation of the water signal during the relaxation delay and mixing time. A 1D 13C spectrum was recorded using an inverse-gated pulse sequence (zgig). Two-dimensional (2D) experiments consisted of a phase-sensitive correlation spectroscopy (COSY) (cosydfgpph19) with a 3-9-19 pulse sequence for water suppression. The Heteronuclear Single Quantum Coherence (HSQC) spectrum was recorded with a phase-sensitive version using Echo/Antiecho-TPPI gradient selection, decoupling during acquisition, and on-resonance presaturation during the relaxation delay (hsqcetgpprsisp2.2). The Heteronuclear Multiple Bond Correlation (HMBC) spectrum was recorded using a sequence with a single low-pass j-filter, gradients for pathway selection, on-resonance presaturation during the relaxation delay, and no decoupling during acquisition (hmbcgplpndprqf). The detailed acquisition parameters can be found in Supplementary Table S1.

### Cell culture

Mouse HL-1 cells were cultivated in Claycomb Medium (Sigma-Aldrich) supplemented with 100 U/ml penicillin, 100 mg/ml streptomycin, 10% FCS, 2 mM L-glutamine, and 0.1 mM norepinephrine at 37°C and 5% CO_2_. Cells were split at 90% confluency into gelatine/fibronectin-coated cell culture flasks.

### MTT assay

The colorimetric MTT assay based on the reduction of MTT to formazan indirectly serves as a readout of overall cellular metabolic activity (due to changes in cell viability, proliferation, or altered mitochondrial capacity) and was therefore used for screening potential adverse effects of mycophenolic acid (MPA) and mycophenolic acid-β-glucuronide (MPA-G) on cardiomyocytes. HL-1 cells were seeded in gelatin/fibronectin-coated 96-well plates and treated for 24 h with MPA (Sigma-Aldrich, dissolved in H_2_O) or MPA-G (Santa Cruz, dissolved in H_2_O) before incubation with MTT (0.5 mg/ml MTT in PBS; Sigma-Aldrich) in phenol-free Dulbecco's Modified Eagle Medium (DMEM) (Sigma-Aldrich) for 10 min, following which formazan generation was measured by absorbance according to the manufacturer's instructions. Data were normalized to the control cells.

### Analysis of apoptosis and necrosis

For the evaluation of apoptosis and necrosis in MPA-G- or MPA-treated cells, HL-1 cells were grown in a gelatin/fibronectin-coated plate and stimulated with different concentrations of MPA-G (Santa Cruz, dissolved in H_2_O; 100–500 µg/ml) or MPA (Sigma-Aldrich, dissolved in H_2_O; 0.5–500 µg/ml) in Claycomb Medium for 24 h. Subsequently, the cells were stained with propidium iodide (Sigma-Aldrich; 1:20) and Annexin V (BD Pharmingen; 1:2,500) for 15 min. Fluorescence was detected via flow cytometry (FACSCanto II, BD Biosciences) and data analysis was performed by using FlowJo™ Software (BD Biosciences), calculating the apoptotic cells (Annexin V^+^) and necrotic cells (propidium iodide^+^) as percentages of all cells.

### Seahorse assay

For analysis of mitochondrial activity, HL-1 cells were plated in gelatin/fibronectin-coated Seahorse XF96 CELL CULTURE Microplates (Agilent) and incubated for 16 h with MPA-G (Santa Cruz) or MPA (Sigma-Aldrich). Before measurement, the medium was changed to phenol-free DMEM supplemented with 4.5-g/L glucose, 2-mM sodium pyruvate, and 2-mM glutamine. The oxygen consumption rate (OCR) was measured under basal conditions and in response to 1 μM oligomycin, 0.3 μM carbonyl cyanide *p*-trifluoromethoxyphenylhydrazone (FCCP), and 1 μM rotenone plus 1 μM antimycin using the XF96 Extracellular Flux Analyzer (Seahorse Bioscience) following the manufacturer's instructions. Data were normalized within each experiment to control cells at the starting point and effects on basal respiration, maximal respiration, and adenosine triphosphate (ATP) production were calculated.

### MitoSOX assay

For measurement of mitochondrial reactive oxygen species (ROS) production, a MitoSOX assay was performed with HL-1 cells grown in a gelatin/fibronectin-coated plate. The cells were stimulated with different concentrations of MPA-G (100–500 µg/ml; Santa Cruz) or MPA (0.5–500 µg/ml; Sigma-Aldrich) in Claycomb Medium. After 24 h, the cells were detached and stained with 1 µM MitoSOX™ Red Mitochondrial Superoxide Indicator (Thermo-Fisher) for 30 min. Subsequently, fluorescence was detected using flow cytometry (FACSCanto II, BD Biosciences) and data analysis was performed by using FlowJo™ Software (BD Biosciences), calculating ROS^+^ cells (PE^+^ cells indicated by gray lines) as percentage of all cells.

### Statistics

Data are shown as mean ± standard deviation (SD). All statistical analyses were performed by using Graph Pad Prism (Version 10, GraphPad Software). Outliers were identified via the Grubbs outlier test. Testing for normal data distribution was performed using the Shapiro–Wilk test. For multiple group comparisons, *P*-values were calculated using (repeated-measures) one-way ANOVA with Dunnett's multiple comparisons test (for parametric data), or using the Friedman test with Dunn's multiple comparisons test (for non-parametric data). For multiple group comparisons to a fixed reference value, a one-sample *t*-test with Bonferroni–Holm correction for multiple group comparison was applied, as appropriate. A *P*-value of ≤0.05 was considered statistically significant (*P < 0.05, **P < 0.01, ***P < 0.001, ****P < 0.0001).

## Results

### Isolation of dialysate fractions negatively affecting overall cardiomyocyte metabolic activity

**Figure 1:**
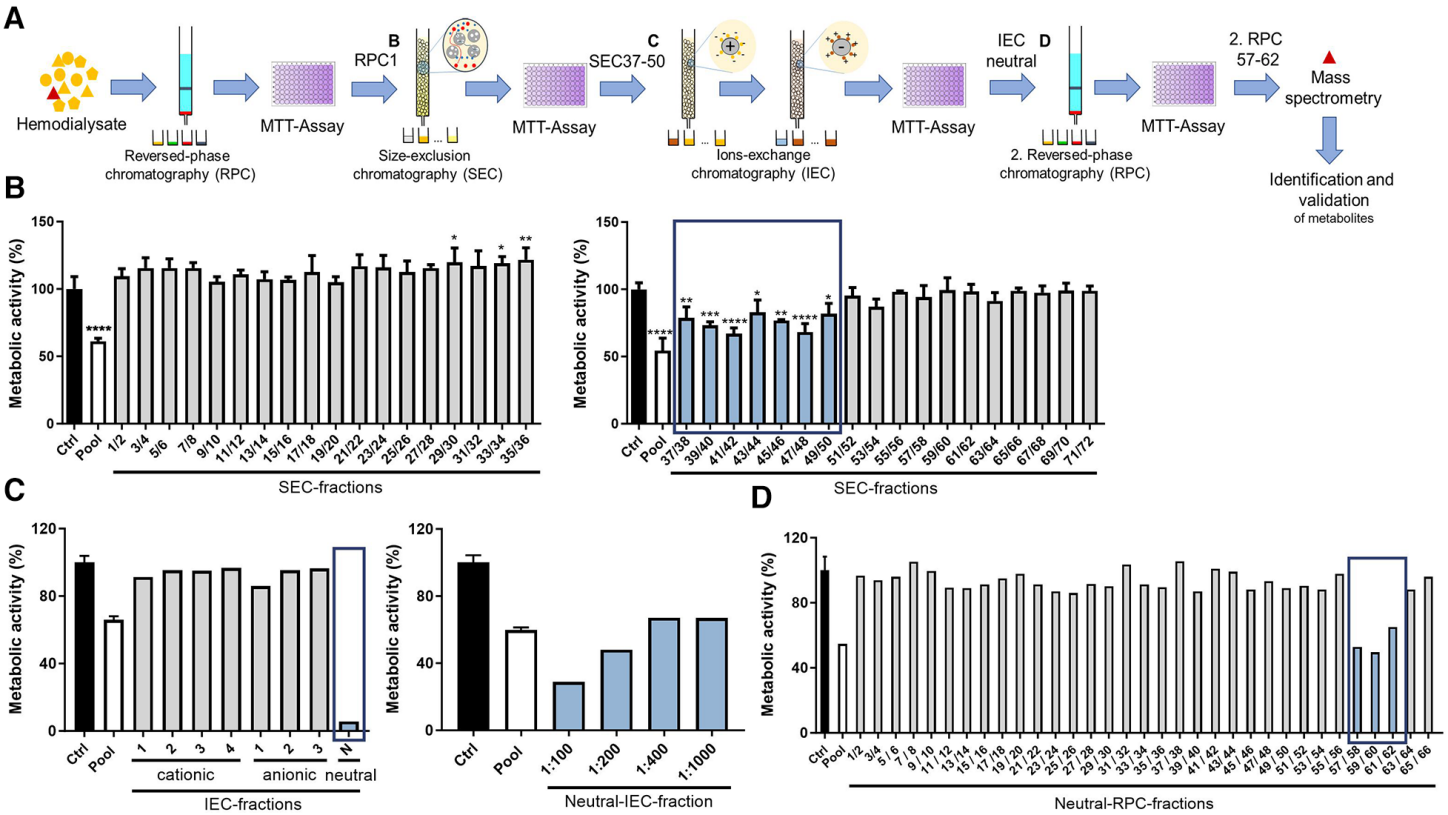
Chromatographic fractionation of dialysate and screening of fractions for effects on overall cardiomyocyte metabolic activity. (**A**) Schematic overview of stepwise approach, different chromatographic steps, and screening procedure. Overall metabolic activity (as % to control) measured using an MTT assay of cardiomyocytes treated with dialysate fractions obtained through (**B**) SEC, (**C**) IEC including dose response of neutral fraction, as well as (**D**) second RPC with continuous gradient. Values are means or means ± SD; each screening step was performed once and when allowed by available fraction volume, in triplicate (**B**) *P < 0.05, **P < 0.01, ***P < 0.001, ****P < 0.0001 using one-way ANOVA with Dunnett's multiple comparisons test comparing each fraction to Ctrl (**B**) Ctrl = vehicle-treated control cells.

To identify novel, potentially harmful substances accumulating in patients with advanced CKD, ∼500 L hemodialysis dialysate was collected from patients with CKD stage 5 and concentrated by reversed-phase chromatography. Using orthogonal chromatography, the dialysate pool was fractionated, with each fraction screened for a potential negative impact on the overall metabolic activity of HL-1 cardiomyocytes using an MTT assay to enable cellular health monitoring in high-throughput format (Figure 1A). First, the dialysate pool was concentrated and fractionated using preparative RPC with stepwise gradient, resulting in four fractions separated based on hydrophilicity. The first hydrophilic fraction (RPC1), but not the other fractions, markedly decreased overall metabolic activity of the cardiomyocytes compared with an unstimulated control after treatment for 24 h (Supplementary Figure S1A). Hence, this fraction RPC1 was further fractionated into 72 fractions based on size using SEC, pooled in groups of two fractions and screened in an MTT assay. Treatment with SEC fractions 37–50 significantly reduced overall metabolic activity of cardiomyocytes compared with the vehicle control (Figure 1B). These specific fractions were confirmed to be endotoxin-negative [lipopolysaccharide (LPS) < 9.5 × 10^−4 ^µg/ml], being concentrations confirmed to not impact readouts in the applied bioassay (Supplementary Figure S1B). A third chromatographic fractionation applied two ion-exchange chromatography (IEC) steps. First, SEC fractions 37–50 were pooled and fractionated into three anionic fractions using anion-exchange chromatography and, second, the flow-through was separated in four cationic fractions using cation-exchange chromatography. The flow-through was collected as a neutral IEC fraction. The treatment of cardiomyocytes with the neutral IEC fraction led to a strong reduction of overall metabolic activity, which was confirmed in a dose–response experiment (Figure 1C). Consequently, the neutral IEC fraction was further fractionated by a second reversed-phase chromatography with continuous gradient into 66 neutral RPC fractions. Of these, fractions 57–62 again decreased overall cardiomyocyte metabolic activity (Figure 1D), indicating the presence of substances with adverse effect on cardiomyocyte health.

### Substance identification in dialysate fraction with negative impact on cardiomyocyte health

**Figure 2:**
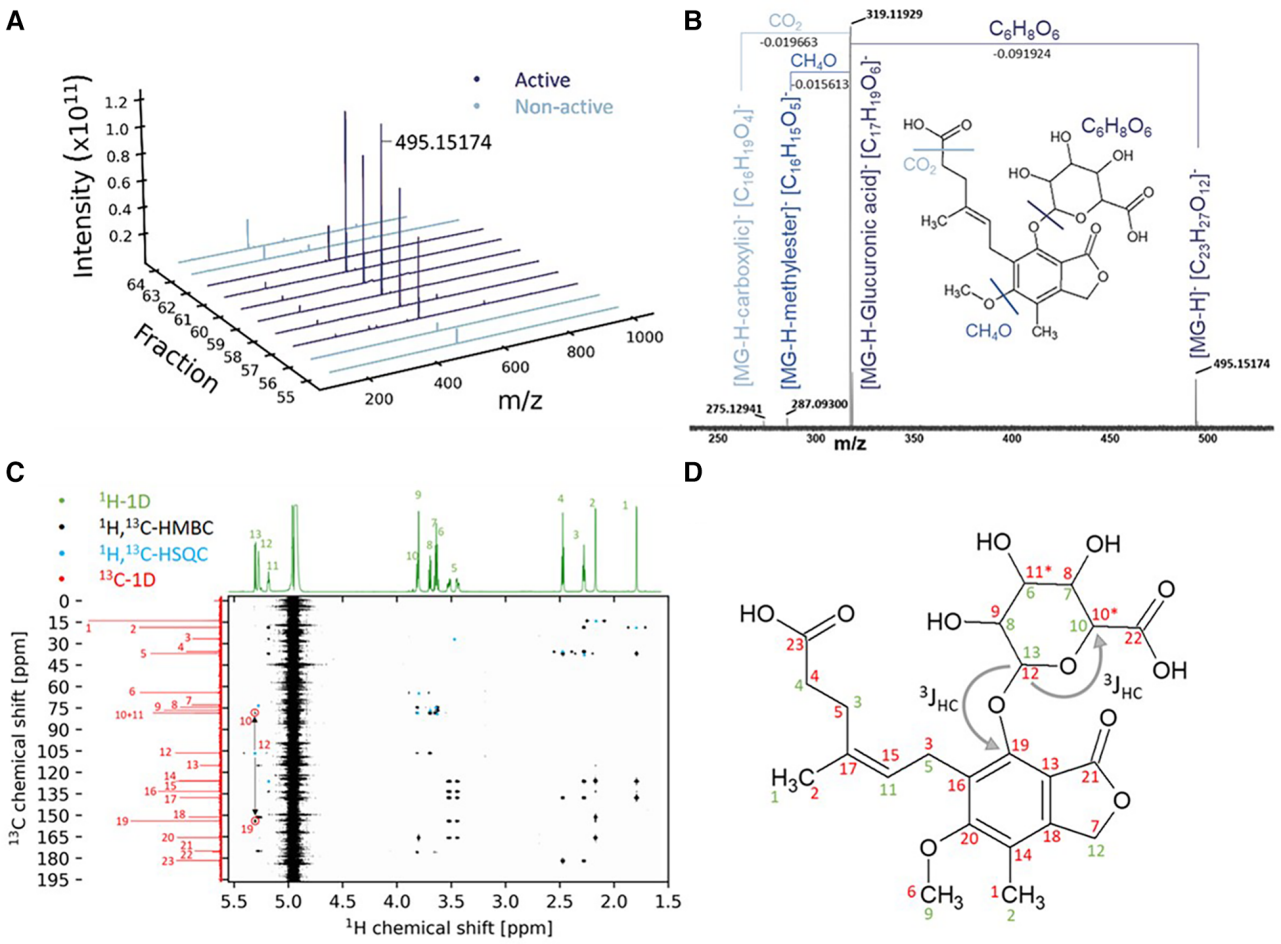
Identification of mycophenolic acid-β-glucuronide using mass spectrometry and nuclear magnetic resonance. (**A**) Highest abundance of the m/z 495.15174 in the bioactive fractions. (**B**) FTICR-MS fragmentation at 7.5 eV CID. (**C**) Combined NMR experiments for structure elucidation of the bioactive fraction. (**D**) Structure of the compound with the annotated NMR-chemical shifts from (**C**) in 1H (green) and 13C (red).

To identify potential metabolites within the bioactive fraction that could be responsible for the observed effect on cardiomyocyte health, we proceeded with direct injection electrospray ionization (ESI)-FTICR-MS of the bioactive and inactive chromatographic fractions to enable the identification of the contained metabolites. This revealed specifically in all bioactive fractions reducing cardiomyocyte health (neutral RPC fractions 57–62), but not in the neighboring inactive fractions, the presence of one single dominant peak with m/z 495.15174 (Figure 2A). The signal was isolated and fragmented in collision-induced dissociation with 2, 5, and 7.5 eV, showing a neutral mass loss of a sugar unit (C_6_H_8_O_6_) (Figure 2B). Through comparison with the human metabolome database (HMDB), the dominant peak at m/z 495.15174 was identified as ID HMDB0130142, involving a main fragment in tandem mass spectrometry of m/z 319.1187 corresponding to the main fragment within our FTICR-MS fragmentation spectrum with m/z 319.1193 and with the formula [C_17_H_19_O_6_]^−^, i.e., the drug MPA. Combined, for the mediator in our bioactive fraction, this led to the putative structure of MPA-G, a metabolite of MPA (Figure 2B). Identification was independently confirmed using NMR, with detailed acquisition parameters and obtained chemical shifts provided in Supplementary Tables S1, S2. The analysis by NMR confirmed this exact structure of the putative annotated compound (Figures 2C,D). All hydrogen and carbon signals were assigned and connectivities established. The still ambiguous question of the sugar moiety position was also solved by the detected long range (^3^J_HC_) couplings between carbon 19, belonging to the aromatic ring, and carbon 12, belonging to the sugar moiety (Figures 2C,D).

### MPA-G and MPA treatment leads to decreased overall metabolic activity and increased apoptosis of cardiomyocytes

**Figure 3:**
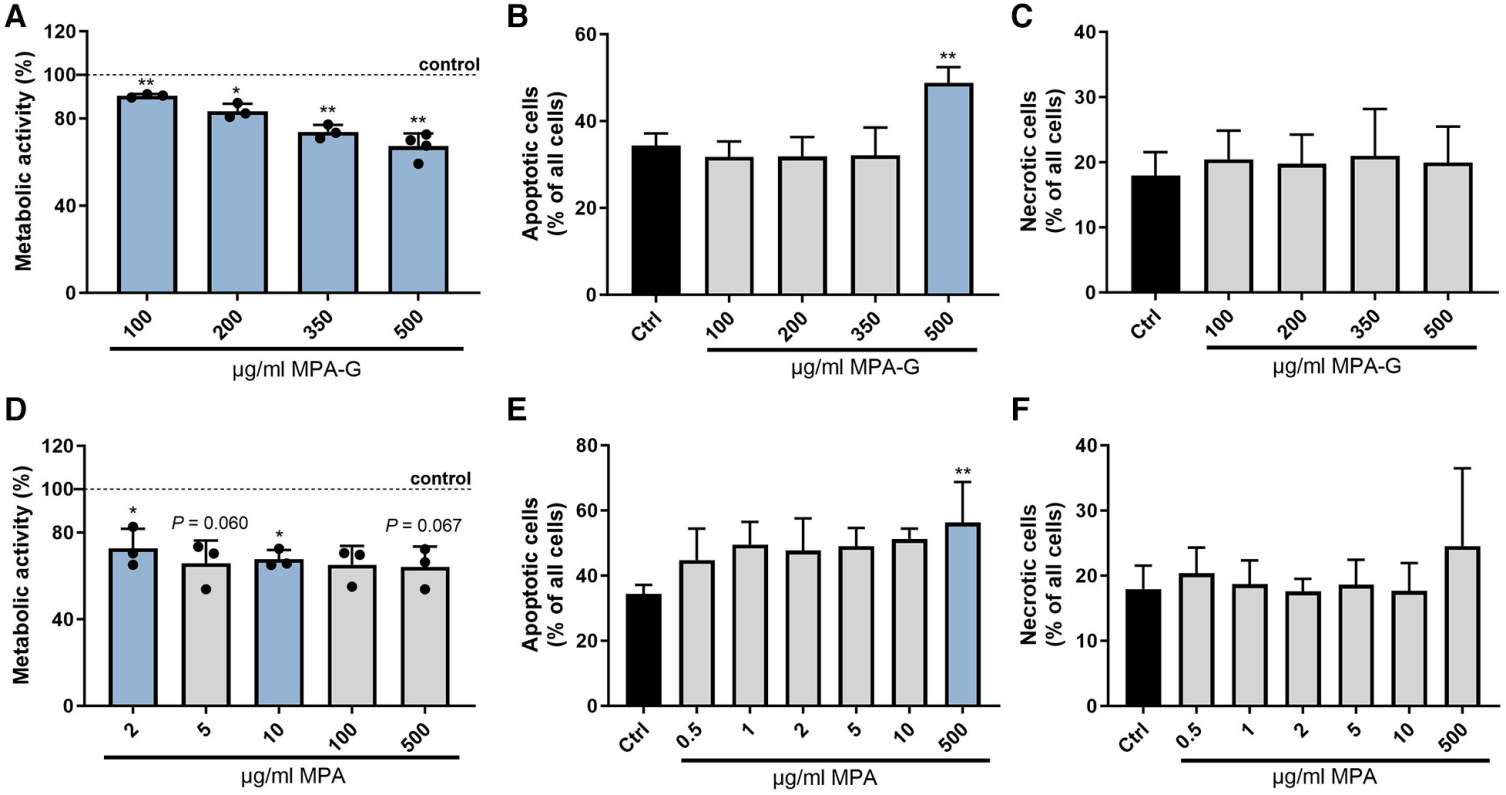
Characterization of effects of MPA-G and MPA on cardiomyocyte viability. Effects of different concentrations of MPA-G or MPA on cardiomyocytes in terms of (**A,D**) overall metabolic activity via the MTT assay (as % relative to control cells), (**B,E**) apoptosis (as % of all cells), and (**C,F**) necrosis (as % of all cells). Values are means ± SD; *n* = 3–4 [except *n* = 2 for (**D**): MPA 0.01/0.05/0.5 µg/ml]; *P < 0.05, **P < 0.01 comparing each MPA-G/MPA concentration with untreated control cells (Ctrl). One-sample *t*-test with Bonferroni–Holm correction for multiple group comparison to a fixed reference value (Ctrl) (**A,D**); repeated-measures One-way ANOVA with Dunnett's multiple comparisons test (**B,F**) or Friedman test with Dunn's multiple comparisons test (**C,E**).

To verify that MPA-G is indeed the mediator found in the dialysate of patients with CKD negatively affecting cardiomyocytes, the overall metabolic activity and cell viability of HL-1 cells was assessed applying different concentrations of MPA-G. MPA-G was tested in the range of 100–500 µg/ml, covering the concentration range previously reported in a dialysis patient on regular dosing with mycophenolate mofetil (MMF) (16)—the prodrug of MPA—as well as in patients on MMF with post-transplant acute kidney failure (17). In comparison, effects of the drug MPA were tested at 100–500 µg/ml, enabling a comparison with comparable MPA-G dosing, but also concentrations down to 0.01 µg/ml were tested, covering the lower range of MPA levels reported in patients with CKD on MMF treatment (16–18). Concentrations of 100–500 µg/ml MPA-G decreased the overall metabolic activity of cardiomyocytes in a dose-dependent way (Figure 3A). Additional Annexin V and propidium iodide staining showed an increased amount of apoptotic cardiomyocytes only after treatment with 500 µg/ml MPA-G, whereas there was no effect of concentrations from 100 to 350 µg/ml MPA-G on apoptosis (Figure 3B) and no effect of MPA-G on necrosis was observed either (Figure 3C). Treatment of cardiomyocytes with concentrations down to 2-µg/ml MPA also led to a significant reduction in metabolic activity (Figure 3D, Supplementary Figure S2). Only the highest MPA concentration could induce a statistically significant increase of cardiomyocyte apoptosis (Figure 3E), but not of necrosis (Figure 3F).

### MPA-G and MPA treatment causes reduced overall mitochondrial respiration capacity and elevated mitochondrial ROS production in cardiomyocytes

**Figure 4:**
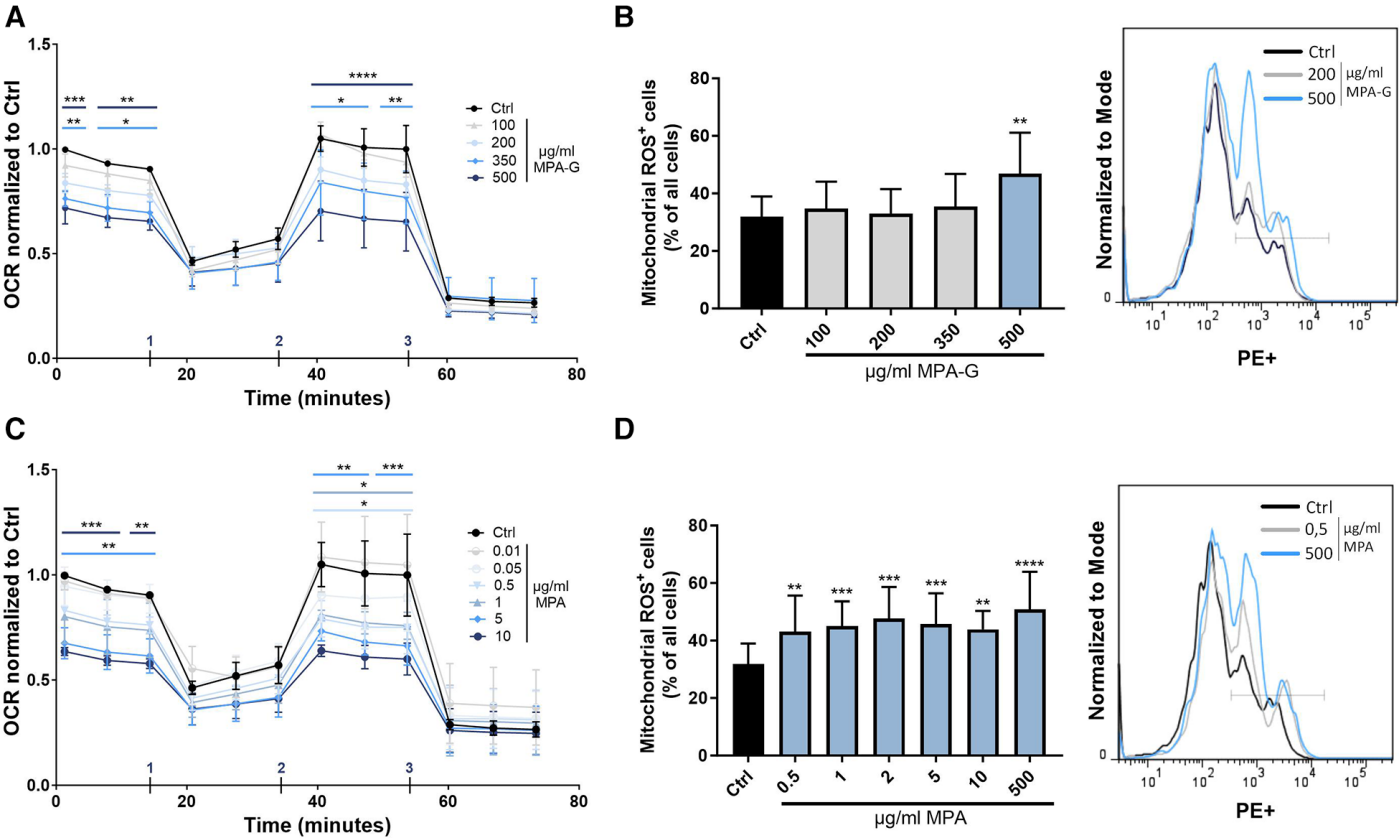
Characterization of effects of MPA-G and MPA on overall mitochondrial function of cardiomyocytes. (**A,B**) Effects of different concentrations of MPA-G or MPA on cardiomyocytes in terms of (**A,C**) OCR (normalized within each experiment to control cells at the starting point) and (**B,D**) mitochondrial ROS production, displayed as ROS-positive cells (as % of all cells) and with representative histogram (with ROS detected in the PE channel). (**A,C**) Addition of (1) oligomycin, (2) FCCP, and (3) Rotenone + antimycin during measurement is indicated. (**A,D**) Values are means ± SD; *n* = 3–4; *P < 0.05, **P < 0.01, ***P < 0.001, ****P < 0.0001 using two-way ANOVA with Dunnett's multiple comparisons test (**A,C**) or repeated-measures One-way ANOVA with Dunnett's multiple comparisons test (**B,D**) comparing each MPA-G/MPA concentration to untreated control cells (Ctrl).

A Seahorse assay for analysis of mitochondrial function showed that MPA-G concentrations of 350–500 µg/ml reduced overall basal and maximal cellular respiration in cardiomyocytes (Figure 4A, Supplementary Figures S3A–C). Mitochondrial ROS production was measured using the MitoSOX™ Red Mitochondrial Superoxide Indicator for live-cell imaging, revealing increased ROS production in cardiomyocytes after treatment with 500 µg/ml MPA-G (Figure 4B). Concentrations of 5 µg/ml MPA and above decreased basal and maximal respiration, whereas lower concentrations of 0.5–1 µg/ml MPA only significantly reduced maximal cellular respiration (Figure 4C, Supplementary Figures S3D–F). Mitochondrial ROS production was increased in response to 0.5–500 µg/ml MPA (Figure 4D). Of note, the analyzed concentration ranges did not affect the pH of the culture medium, excluding pH-dependent effects.

## Discussion

CKD is known to increase cardiovascular risk as well as mortality, with CKD being an independent risk factor for cardiovascular events (5). Overall, during our screening for novel substances accumulating in CKD patients with adverse effects on cardiovascular health, we identified the MMF drug metabolite MPA-G as affecting cardiomyocyte health at concentrations specifically found in patients with kidney failure treated with MMF, reflected by impaired overall cardiomyocyte metabolic activity and mitochondrial respiration capacity as well as increased mitochondrial ROS production.

In general, CKD patients receive a mean of 12 different medications to treat their underlying kidney disease and a large number of associated comorbidities (19). Impaired kidney excretion of drugs is well documented in patients with CKD (19–21). Furthermore, significant changes also occur in hepatic drug metabolism in CKD (19, 21) owing to the alterations in the expression and activity of drug-metabolizing enzymes and transporters localized in the liver (22, 23), collectively promoting systemic accumulation of drugs and potentially toxic drug metabolites in patients with CKD (20).

The MMF drug metabolite MPA-G is metabolized from the pharmacologically active MPA (24), which is ingested as its prodrug MMF and is hydrolyzed as well as absorbed in the stomach. MPA acts as a potent inhibitor of inosine monophosphate dehydrogenase type 2 (IMPDH-2) and thus of *de novo* guanine nucleotide synthesis. With lymphocytes mainly depending on this pathway instead of the salvage pathway for guanine production, MMF has been used to induce a relatively selective targeting of lymphocyte proliferation, enabling immunosuppression in patients prepared for or after solid organ transplantation (25, 26). In this context, MMF is widely used in kidney allograft recipients (27–29). In addition, it is also used for the treatment of autoimmune and non-transplant kidney diseases (30).

Within minutes after application, MMF is de-esterified to MPA (25). MPA in its turn is rapidly metabolized by UDP-glucuronosyl transferase enzymes in the kidney, liver, and gut to MPA-G, which is not pharmacologically active and up to 87% of which is excreted via urine, underlining the importance of the kidney function in drug excretion (25, 31). Healthy volunteers treated with a single dose of 1-g MMF displayed a maximal MPA-G concentration of 28.0–31.4 µg/ml 1.3–1.7 h post-administration and a half-life time of 12.5–15.2 h (26). However, studies revealed a reduced MPA-G clearance with decreasing kidney filtration function (26), as well as a high accumulation of MPA-G in plasma of dialysis patients upon MMF treatment. For example, a dialysis patient treated with a relatively low daily dose of 750-mg MMF (250 mg in the morning and 500 mg in the evening) for 4 weeks displayed MPA-G levels of 120 µg/ml even prior to daily MMF intake and of 142 µg/ml 2 h after the first 250 mg intake (16). Also, dialysis patients with a single dose of 1-g MMF showed a very slow clearance of MPA-G with an area under the curve (AUC) of 1,565 µg/ml h being five times as much as expected in subjects with normal kidney function (16). Within 12 months after kidney transplantation, patients revealed MPA-G levels of 2.8–84.3 µg/ml, with highest concentrations for lowest GFR values (18) and with high MPA-G levels up to 358 µg/ml detected in patients with post-transplant acute kidney failure (17). These concentrations are very close, respectively, within the range in which MPA-G adversely affected cardiomyocyte health in our assays (100–500 µg/ml). Here, MPA-G was validated to dose-dependently decrease the overall metabolic activity as well as basal and maximal respiratory capacity of cardiomyocytes at concentrations relevant for patients with failing kidney function treated with MMF. Mitochondria as the power house of the cell play an important role in cardiomyocytes because of the enormous energy requirements of cardiac contractions (32). Furthermore, mitochondrial dysfunction is associated with cardiac dysfunction (33). Therefore, our findings alert to potential adverse effects of the MMF metabolite MPA-G on cardiomyocyte health upon long-term treatment specifically in patients with kidney failure due to an abrogated kidney-mediated clearance and resulting high systemic accumulation of MPA-G. This is especially the case when MMF is given at high doses and over a longer term, taking into account a potential further accumulation of MPA-G with plasma concentrations at 20–100-fold higher levels compared with the parent compound (34) as well as the already highly increased cardiovascular risk of patients in advanced stage of kidney damage (5).

Compared with MPA-G, MPA showed a faster clearance from the circulation (16) and no to minimal effect of kidney dysfunction on the plasma AUC of MPA (26), although variable maximal plasma levels have been reported among different studies. For example, one study only found MPA-G but not MPA to accumulate in patients with post-transplant acute kidney failure treated daily with 3-g MMF, reporting MPA levels in the range of 0.5–2.3 µg/ml (17). Others reported maximal levels of MPA in the range of 5.1–16.0 μg/ml in hemodialysis patients 0.66 h after intake of 0.5–1 g MMF (16); concentrations within 0.2–10.4 µg/ml in patients up to 12 months after kidney transplantation (18), or even levels rising to ∼20–30 µg/ml in patients following solid organ transplant dosed 1–2 g MMF/day (25).

For MPA, anti-inflammatory and anti-oxidative effects have previously been shown in primary vascular smooth muscle cells (VSMCs), fibroblasts, and endothelial cells. MPA concentrations above 10 nM (∼0.0032 µg/ml) inhibited platelet-derived growth factor (PDGF)-induced cellular ROS formation in primary rat VSMCs (35) and interfered with O_2_^−^ formation in primary human endothelial cells (HUVECs) by blocking basal activity of endothelial NAD(P)H oxidase (36). In primary as well as immortalized mouse fibroblasts, 5 µM MPA reduced inflammation by decreasing interleukin-6 (IL-6) production (37). By contrast, we detected reducing effects of MPA on the overall metabolic activity and basal mitochondrial function of HL-1 cells at concentrations of 2–5 µg/ml (∼6–15 µM) and above. In the same line, others have reported a negative impact of MPA on primary VSMC, endothelial, and fibroblast proliferation and migration at even lower concentrations (≥0.1 µM = 0.032 µg/ml) (38, 39) as well as on proliferation (0.031 µg/ml MPA) and differentiation (0.125 µg/ml MPA) of murine embryonic stem cells (40). Also, further studies have proved an embryotoxic potential of MPA causing a reclassification of the immunosuppressant as category D comprising drugs with evidence of fetal risk (41). Similarly, anti-proliferative and pro-apoptotic effects of MPA were shown in tumor cells from low concentrations of (IC50 < 0.5 µg/ml) (42), and it was suggested that MPA might interfere with a nuclear function of IMPDH in repressing E2F as a master regulator of cell cycling (43, 44).

However, it needs to be taken into account that, in contrast to MPA-G, high MPA levels in blood are mainly a temporary effect with maximal MPA levels detected only shortly after MMF intake (16). Thus, although in hemodialysis patients, transplant patients as well as healthy volunteers on MMF treatment, maximal plasma concentrations of MPA have been reported that are above the MPA concentrations negatively impacting on HL-1 cardiomyocyte health in our assays (16, 18, 25, 26, 45), these are only temporary effects. In hemodialysis patients hemodialysis, maximal MPA concentrations of up to 16 µg/ml shortly after intake of a single oral dose of 1-g MMF rapidly declined to steady levels below 2 µg/ml 3–36 h after MMF intake (16). Conversely, an MPA target AUC_0–12 h_ of 30–60 mg·h/L in transplant patients in the presence of a calcineurin inhibitor (25) would equal 2.5–5 µg/ml in case of steady dosing, hence being just within the observed concentration range that negatively impacts cardiomyocytes. Also, MMF has increasingly been used for the treatment of, for example, children with the autoimmune disease systemic lupus erythematosus, with patient subsets reported to display MPA plasma levels above 2 µg/ml even up to 8 h after MMF intake and with an MPA target AUC_0–12 h_ also >45 mg h/L (46). Therefore, overall, a comparative reflection of MPA effects on diverse cell types *in vitro* as well as a closer reflection of individual MMF dosing in terms of an accumulation of MPA, and especially also MPA-G, would be interesting, both in patients with kidney failure and beyond.

Previous studies detected MPA-G in fluids of hemodialysis as well as peritoneal dialysis in MMF-treated patients with end-stage kidney failure (16) or after kidney transplantation (47). Nonetheless, removal of MPA-G from the circulation by hemofiltration is five times slower compared with the excretion expected by healthy kidneys (16), explaining why MPA-G accumulates in patients with kidney failure despite regular dialysis. As an underlying cause, ∼82% of MPA-G was shown to be protein-bound (26), with hemodialysis less efficiently removing protein-bound substances from the circulation, as for example shown for indoxyl sulfate and phenyl acetic acid (48, 49). Again, this warrants for MPA-G accumulation in MMF-treated patients with kidney failure despite regular dialysis. As a limitation of our study, a differential delivery of the substance to cardiomyocytes *in vitro* vs. *in vivo* may impact its ultimate cellular effect. Also, we did not examine the differential dosage effects of protein-bound vs. free MPA-G on cardiomyocytes, and—by not supplementing additional albumin up to levels reached in the blood in our *in vitro* experiments—we may be overestimating the effect of MPA-G. Conversely, *in vivo* the substances have a much longer contact time with the cells compared with our *in vitro* setting and to date it also remains unclear whether only the free forms or also the protein-bound substances can cause cellular effects. As another limitation, we measured in our mitochondrial metabolic analyses the overall mitochondrial respiration capacity without a specific focus on long-chain fatty acid usage as the main substrate (60%–90%) for cardiomyocyte energy production. These aspects require further investigation in a follow-up study, with also an additional in-depth investigation on the underlying molecular mechanisms of MPA-G-induced mitochondrial effects and a comparison of animal models with healthy vs. markedly reduced kidney function.

In summary, pharmacokinetics of the drug MMF and its metabolites MPA and MPA-G is mostly described in healthy subjects with increasing information available also on patients with CKD, but side effects of MPA and its metabolite MPA-G have rarely been studied. In this paper, MPA-G was discovered to exert an adverse effect on cardiomyocyte health at concentrations found in patients with kidney failure. Hence, considering that patients having reduced kidney function already display increased cardiovascular risks, this study points to the potential adverse effects of long-term high MMF dosing in such patients. Future studies should address the effects of MPA-G on cardiovascular health in more detail using *in vivo* models.

## Data Availability

The original contributions presented in the study are included in the paper/Supplementary Material, further inquiries can be directed to the corresponding author.

## Supplementary material

Supplementary Figure S1, Supplementary Figure S2, Supplementary Figure S3, Supplementary Table S1, Supplementary Table S2 Analyses of effects on overall metabolic activity of cardiomyocytes. (**A,B**) Overall metabolic activity measured using an MTT assay of cardiomyocytes treated (**A**) with dialysate fractions obtained through reversed-phase chromatography (RPC) with continuous gradient or pool or (**B**) with lipopolysaccharide (LPS) in comparison with unstimulated cells. (**A,B**) Unstimulated controls within each independent experiment were set to 100%, as indicated by the horizontal lines. Shown are means ± SD (*n *= 3). ***P *< 0.01, ***P *< 0.01 using one-sample *t*-test or Wilcoxon test with Bonferroni–Holm correction for multiple group comparison to a fixed reference value (Control cells). Characterization of effects of MPA on cardiomyocyte viability. Effects of different concentrations of MPA on cardiomyocytes in terms of overall metabolic activity (as % relative to control cells). Unstimulated control cells within each independent experiment were set to 100%, as indicated by the horizontal line. Shown are means ± SD; *n *= 2–3, as indicated. One-sample *t*-test with Bonferroni–Holm correction for multiple group comparison to a fixed reference value (Control cells). Characterization of effects of MPA and MPA-G on mitochondrial activity of cardiomyocytes. Effects of different concentrations of MPA-G or MPA on the oxygen consumption rate of cardiomyocytes (OCR, normalized within each experiment to control cells at the starting point, resulting in relative values, as shown in Figures 4A,C), followed by the quantification of the following phases: (**A,D**) Basal respiration; (**B,E**) Maximal respiration; and (**C,F**) ATP production. (**A–F**) Shown are means ± SD; *n *= 3; **P *< 0.05, ***P *< 0.01, for comparison of each MPA-G/MPA concentration to unstimulated control cells (unst). Repeated-measures one-way ANOVA with Dunnett’s multiple comparisons test (**A–C,E–F**) or Friedman test with Dunn’s multiple comparisons test (**D**). Detailed acquisition parameters of the NMR analysis. Detailed chemical shifts obtained in the NMR analysis of the bioactive fraction.
