# Supplementary material for: Increased levels of a mycophenolic acid metabolite in patients with kidney failure negatively affect cardiomyocyte health
Source: Front Cardiovasc Med. 2024 Mar 6;11:1346475. doi: 10.3389/fcvm.2024.1346475 (PMC10951386; doi:10.3389/fcvm.2024.1346475)
Supplement: Supplementary Figure S1, Supplementary Figure S2, Supplementary Figure S3, Supplementary Table S1, Supplementary Table S2 — Analyses of effects on overall metabolic activity of cardiomyocytes. (A,B) Overall metabolic activity measured using an MTT assay of cardiomyocytes treated (A) with dialysate fractions obtained through reversed-phase chromatography (RPC) with continuous gradient or pool or (B) with lipopolysaccharide (LPS) in comparison with unstimulated cells. (A,B) Unstimulated controls within each independent experiment were set to 100%, as indicated by the horizontal lines. Shown are means ± SD (n = 3). **P < 0.01, **P < 0.01 using one-sample t-test or Wilcoxon test with Bonferroni–Holm correction for multiple group comparison to a fixed reference value (Control cells). Characterization of effects of MPA on cardiomyocyte viability. Effects of different concentrations of MPA on cardiomyocytes in terms of overall metabolic activity (as % relative to control cells). Unstimulated control cells within each independent experiment were set to 100%, as indicated by the horizontal line. Shown are means ± SD; n = 2–3, as indicated. One-sample t-test with Bonferroni–Holm correction for multiple group comparison to a fixed reference value (Control cells). Characterization of effects of MPA and MPA-G on mitochondrial activity of cardiomyocytes. Effects of different concentrations of MPA-G or MPA on the oxygen consumption rate of cardiomyocytes (OCR, normalized within each experiment to control cells at the starting point, resulting in relative values, as shown in Figures 4A,C), followed by the quantification of the following phases: (A,D) Basal respiration; (B,E) Maximal respiration; and (C,F) ATP production. (A–F) Shown are means ± SD; n = 3; *P < 0.05, **P < 0.01, for comparison of each MPA-G/MPA concentration to unstimulated control cells (unst). Repeated-measures one-way ANOVA with Dunnett’s multiple comparisons test (A–C,E–F) or Friedman test with Dunn’s multiple comparisons test (D). Detailed acquisition parameters of the NMR analysis. Detailed chemical shifts obtained in the NMR analy [file Datasheet1.pdf]

SUPPLEMENTAL MATERIAL

Supplemental Tables

Suppl. Table 1. Detailed acquisition parameters of the NMR analysis.

Experiment	Bruker library name	AQ (F2)	D1	Mixing time	Scans	Increments	1J(H,C)	longJ(H,C)
1D 1H	noesypr1d	5 s	5 s	10us	32	/	/	/
1D 13C	zgig	2.4 s	12.6 s	/	15392	/	/	/
COSY	cosydfgpph19	1.27 s	0.72 s	/	8	1060	/	/
HSQC	hsqcetgpprsisp2.2	0.25 s	1.25 s	/	16	420	150	/
HMBC	hmbcgplpndprqf	1 s	0.5 s	/	160	327	150	9

Suppl. Table 2. Detailed chemical shifts obtained in the NMR analysis of the bioactive fraction.

Carbon Nr. (Figure 2D)	13C-chemical shift (ppm)	Attached 1H- chemical shift (ppm)
1	13.95	2.17
2	18.61	1.79
3	26.56	3.48
4	35.55	2.47
5	36.88	2.28
6	64.21	3.80
7	72.82	5.27
8	74.45	3.64
9	76.60	3.69
10	78.41	3.81
11	78.46	3.63
12	106.47	5.30
13	115.15	/
14	125.86	/
15	126.21	5.18
16	133.29	/
17	137.66	/
18	151.24	/
19	153.97	/
20	165.65	/
21	174.98	/
22	175.72	/
23	181.75	/

Supplemental Figures

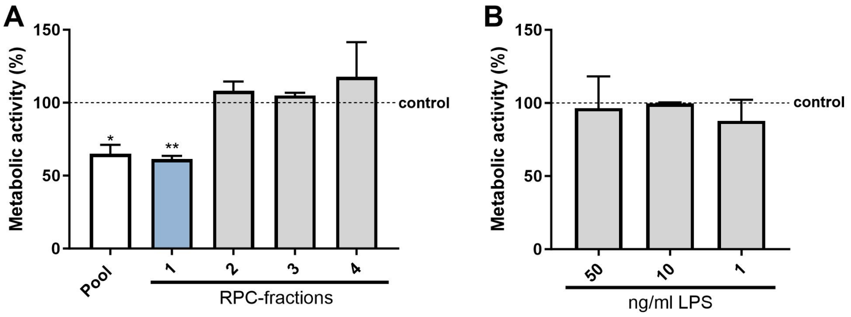

Suppl. Figure 1: Analyses of effects on overall metabolic activity of cardiomyocytes. *(A-B) Overall metabolic activity measured using an MTT assay of cardiomyocytes treated (A) with dialysate fractions obtained through reversed-phase chromatography (RPC) with continuous gradient or pool or (B) with lipopolysaccharide (LPS) in comparison to unstimulated cells. (A, B) Unstimulated controls within each independent experiment were set to 100%, as indicated by the horizontal lines. Shown are means ± SD (n=3). **P<0.01, **P<0.01 using one-sample t-test or Wilcoxon test with Bonferroni-Holm correction for multiple group comparison to a fixed reference value (Control cells).*

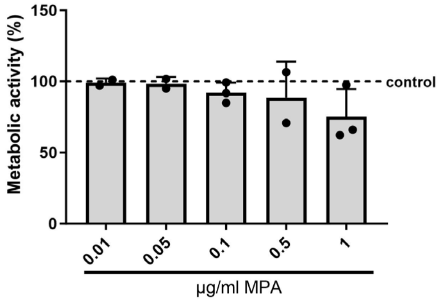

Suppl. Figure 2: Characterization of effects of MPA on cardiomyocyte viability. *Effects of different concentrations of MPA on cardiomyocytes in terms of overall metabolic activity (as % relative to control cells). Unstimulated control cells within each independent experiment were set to 100%, as indicated by the horizontal line. Shown are means ± SD; n=2-3, as indicated. One-sample t-test with Bonferroni-Holm correction for multiple group comparison to a fixed reference value (Control cells).*

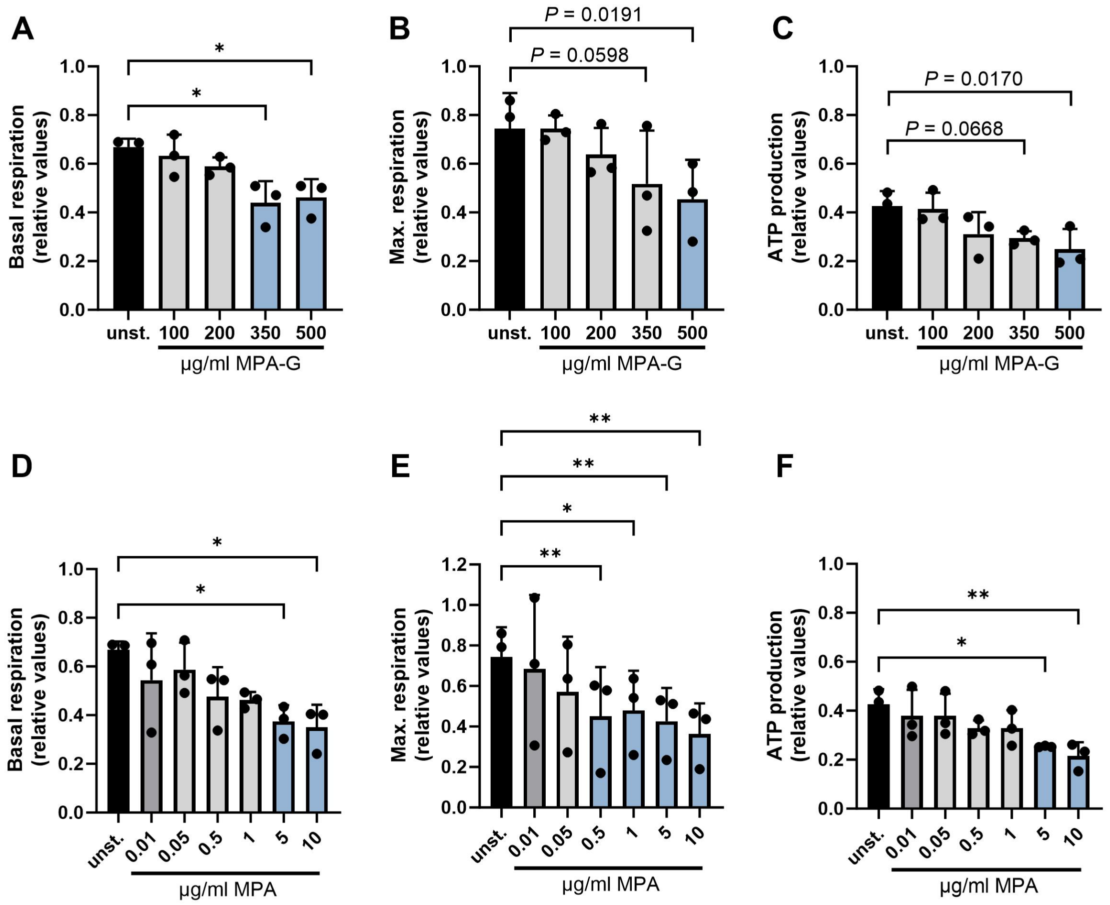

Suppl. Figure 3: Characterization of effects of MPA and MPAG on mitochondrial activity of cardiomyocytes. *Effects of different concentrations of MPA-G or MPA on the oxygen consumption rate of cardiomyocytes (OCR, normalized within each experiment to control cells at the starting point, resulting in relative values, as shown in Figure 4A,C), followed by the quantification of the following phases: (A,D) Basal respiration; (B, E) Maximal respiration; and (C, F) ATP production. (A-F) Shown are means ± SD; n=3; *P<0.05, **P<0.01, for comparison of each MPA-G/MPA concentration to unstimulated control cells (unst). Repeated-measures one-way ANOVA with Dunnett's multiple comparisons test (A-C, E-F) or Friedman test with Dunn's multiple comparisons test (D).*
